# Longitudinal relations between autistic-like features and functional somatic symptoms in adolescence

**DOI:** 10.1177/13623613221143874

**Published:** 2023-01-01

**Authors:** Elske Hogendoorn, Catharina A Hartman, Sarah M Burke, Marijn W G van Dijk, Judith G M Rosmalen

**Affiliations:** University of Groningen, The Netherlands

**Keywords:** adolescence, autistic-like features, functional somatic symptoms, longitudinal

## Abstract

Autistic-like features and functional somatic symptoms (FSS) frequently co-occur. It remains unknown how autistic-like features and FSS affect each other and develop throughout adolescence. This study examined reciprocal relations between autistic-like features and FSS in adolescence. Participants were 2772 adolescents (52.5% male) from the Tracking Adolescents’ Individual Lives Survey population and clinical cohort. Data from four waves were included, covering the ages between 11 and 19 years. Autistic-like features were measured using the Children’s Social Behavior Questionnaire. FSS were assessed using the Youth Self Report and Adult Self Report, respectively. Using the random intercept cross-lagged panel model, a stable positive, moderately strong between-persons association was found between autistic-like features and FSS. No within-persons reciprocal effects from wave to wave were observed. Secondary analyses revealed a consistent relation with FSS for three different domains of autistic-like features (social and communication behaviors, repetitive behaviors, and self-regulatory behaviors), and highly similar interrelations in a subsample of adolescents with a clinical autism spectrum disorder diagnosis. In conclusion, the co-occurrence between autistic-like features and FSS is stable throughout adolescence. Clinicians working with adolescents with autistic-like features should be alert to the presence FSS, and vice versa.

**Lay abstract:**

Adolescents with autistic-like features frequently experience unexplained somatic symptoms too, and vice versa. We followed 2772 adolescents for 8 years, starting at 11 and ending at 19 years of age. At four different moments during this time, we asked these adolescents how often they suffered from unexplained somatic symptoms, such as stomachache and dizziness. We asked their parents to what extent the adolescents showed autistic-like features at those four moments too. Then, we assessed whether the relation between autistic-like features and unexplained somatic symptoms stayed the same between 11 and 19 years old. We also looked at whether there was a reciprocal influence. So far, most studies only looked into the relation between autistic-like features and unexplained somatic symptoms at a specific moment in time. It is important to study how this relation develops over time in adolescence, so we can improve treatment for burdening co-occurring symptoms. In our sample, adolescents who experienced many autistic symptoms also experienced many unexplained somatic symptoms. This relation stayed the same over time. There was no reciprocal influence, so higher autistic-like features did not contribute to higher unexplained somatic symptoms, or the other way around. The findings of this work tell us that in adolescents with autistic-like features it is important to be alert to the presence of unexplained somatic symptoms, and vice versa.

## Introduction

Autism spectrum disorder (ASD) symptoms involve difficulties in social interaction and communication, and restricted and repetitive behavior (American Psychological Association, 2013). A continuum of autistic-like features is present in adolescents in the general population, meaning that adolescents without a clinical diagnosis of ASD also vary to some extent on autistic-like features (Constantino & Todd, 2003). Autistic adolescents are situated at the end of this continuum. ASD is one of the most common developmental disorders and is observed in approximately 6 in 1000 individuals, although estimates vary considerably across countries and range from 0.8 to 93 in 1000 (Chiarotti & Venerosi, 2020).

Autistic-like features are associated with somatic symptoms. Especially gastrointestinal complaints are more prevalent in autistic children and adolescents than in non-autistic peers (Chaidez et al., 2014; Mayes et al., 2021). Meta-analytic evidence shows a greater than threefold elevated risk of constipation, diarrhea, and general gastrointestinal complaints (i.e. bowel complaints or gastrointestinal complaints not otherwise specified) for autistic children aged 2–18 years compared to those without ASD (McElhanon et al., 2014). The same study reported a greater than twofold elevated risk of abdominal pain.

There are indications that other somatic symptoms are also related to autistic-like features. A moderate increase in the prevalence of frequent severe headaches/migraines was found in autistic children, compared to non-autistic children (Gurney et al., 2006). Yet another study showed incontinence and fatigue to be more common in autistic children (Mayes et al., 2021). In preschoolers, a relation between ASD and somatic symptoms was found that remained significant after removing the items concerning gastrointestinal complaints (Fulceri et al., 2016). Other studies reported a significantly higher aggregate of somatic symptoms in autistic children compared to non-autistic peers (Bos et al., 2018; Paul et al., 2015). Another study found higher levels of autistic-like features in a clinical sample of children and adolescents with chronic pain, compared to the general population (Lipsker et al., 2018). This indicates that autistic-like features also often co-occur with somatic symptoms that are unrelated to the gastrointestinal tract, such as headaches or pains elsewhere in the body. Children with a co-occurrence of somatic symptoms show more severe autistic-like features and additional difficulties such as anxiety and externalizing behavior problems, and report a lower quality of life (Aldinger et al., 2015; Fulceri et al., 2016; Kuhlthau et al., 2018).

Explanations for the co-occurrence of autistic-like features and somatic symptoms may be found in the restricted diet often seen in autistic individuals (Sharp et al., 2013) and biomedical mechanisms such as inflammations and altered gut microbiota (Kim et al., 2022). However, often no sufficient explanatory organic cause is found for somatic symptoms, in which case these are referred to as functional somatic symptoms (FSS) (Beck, 2008). Abdominal pain, which is often associated with ASD, is among the most common FSS in children (Domènech-Llaberia et al., 2004).

Autistic-like features and FSS may affect each other in different ways. On the one hand, autistic-like features could contribute to FSS. First, problems in social interaction can lead to anxiety and stress (Spain et al., 2018), which can manifest themselves in somatic symptoms (a process that is called “somatization”) (Beck, 2008). Second, altered sensitivity to sensory stimuli can influence somatic symptom perception. The literature suggests that interoceptive processes occur differently in autistic individuals. Autistic individuals show a reduced ability to accurately detect bodily signals, alongside an increased sensitivity to bodily sensations (Garfinkel et al., 2016). This could contribute to higher reporting of FSS. Third, difficulties in cognitive flexibility may lead to fixation on bodily sensations, which heightens the intensity of somatic symptoms (Hatta et al., 2019).

On the other hand, FSS could contribute to the persistence or exacerbation of autistic-like features by hampering social development. School absenteeism due to FSS leads to less opportunity to practice social skills (Youssef et al., 2006). Although social skills emerge in early childhood, the development of these skills in everyday functioning is an ongoing process, especially of importance during adolescence, when many social changes take place. Moreover, adolescents with FSS often experience stigmatization and feelings of loneliness, which could lead to social rejection and withdrawal (Looper & Kirmayer, 2004; Moulin et al., 2015). Indeed, research has shown that chronically ill adolescents have less social involvement, show more difficulty with making friends and use less adequate conversational skills than healthy peers (Pinquart & Teubert, 2012). In this way, FSS could lead to difficulties in social interaction, which could contribute to persistence or exacerbation of autistic-like features. Undoubtedly, there are shared factors that contribute to both FSS and autistic-like features. These may be factors such as genetic predisposition (Grove et al., 2019; Kato et al., 2010) and other mental health problems (Janssens et al., 2010; Strang et al., 2012). Gaining a better understanding of the interrelations between autistic-like features and FSS can be beneficial to aid in improving healthcare for adolescents with such problems.

Adolescence is a particularly relevant period to examine the intertwined development of autistic-like features and somatic symptoms. This transition period brings along many developmental changes, physical as well as social and emotional, which are also highly relevant for FSS and autistic-like features. While FSS are widespread among young children, most symptoms decrease during adolescence (Janssens et al., 2011). Autistic-like features among adolescents with an ASD diagnosis are relatively stable but may fluctuate across time in the developing adolescent (Bal et al., 2019). Especially in those with milder forms of ASD, developmental improvements may be observed (Horwitz et al., 2020). In general, autistic-like features slightly decrease during adolescence (Seltzer et al., 2004). The developmental pathways of interrelations between autistic-like features and FSS are unknown and age-related patterns of autistic-like features and FSS may also be different if these co-occur. Most available studies are cross-sectional. These used wide age ranges and are, therefore, not informative as to whether associations between autistic-like features and FSS are different across development. Due to these gaps in the literature, a clear developmental perspective for co-occurring autistic-like features with FSS is still missing.

The current study focuses specifically on self-reported FSS, that is, somatic symptoms without known origin (e.g. headache and nausea), in relation to autistic-like features. We adopt a dimensional, trait-based approach of autistic-like features, thus considering a broader construct than the clinical ASD diagnosis. Previous studies have disputed a distinct boundary between clinical ASD and autistic-like features present in the general population (Constantino & Todd, 2003; Lundström et al., 2012). Including the entire variation of autistic-like features can be advantageous in investigating the relation with FSS, so that processes in milder manifestations of symptoms, present in the general population, can also be observed. The aim of this study was to describe developmental pathways of the co-occurrence of autistic-like features and FSS. To this end, we clarified potential reciprocal relations between autistic-like features and FSS, and explored the strength and immediacy of these effects. Based on previous findings suggesting that autistic-like features could affect somatic symptoms, and, conversely, somatic symptoms could also affect autistic-like features, we hypothesized that: (1) There is a consistent and stable cross-sectional positive association between autistic-like features and FSS throughout adolescence, and (2) there is a positive reciprocal relation over time between autistic-like features and FSS during adolescence, beyond the positive cross-sectional associations at the different ages. Using a longitudinal design, we studied a large group of individuals from late childhood (10–12 years) through early adulthood (18–20 years) during four successive assessment waves. We combined data from a general population cohort and a clinical cohort to examine associations between autistic-like features and FSS across the full spectrum of symptom severity.

## Method

### Sample and procedure

This study is part of the TRacking Adolescents’ Individual Lives Survey (TRAILS). Detailed information about sample selection and characteristics has been reported elsewhere (Oldehinkel et al., 2015). In the current study, data from both the population cohort (TRAILS) and the clinical cohort (TRAILS-cc) were used (combined: *n* = 2772; 52.5% male at T1). TRAILS is an ongoing prospective general population cohort study of Dutch adolescents (*n* = 2230 at T1; data from one participant were subsequently deleted upon request of this person, which is a right of participants as part of the ethical procedure). Participants were recruited at age 10–12 years old and were living in the north of the Netherlands at the time of the first assessment wave. TRAILS-cc is a clinical cohort (*n* = 543 at T1) that runs in parallel with TRAILS including the same data collection. Participants from TRAILS-cc were recruited from a large child psychiatric outpatient clinic in the northern Netherlands with the same target area as covered by the population cohort. Children aged 10–12 years who had been referred to this clinic at any point in their life and regarding any type of mental health problem were eligible for participation in TRAILS-cc. Autistic-like features were higher in TRAILS-cc compared to TRAILS. This enabled us to capture a wide range of autistic-like feature severity.

**Figure 1.**
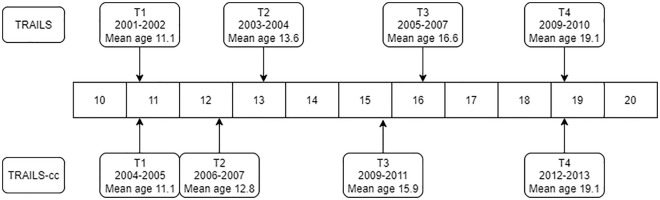
Data collection timeline of TRAILS and TRAILS-cc. Note. The numbers represent the ages in years. TRAILS: TRacking Adolescents’ Individual Lives Survey; TRAILS-cc: TRAILS clinical cohort.

Figure 1 shows the timeline of the data collection and the average ages of the participants at each wave. We used four assessment waves of both samples in the current study.

The study was ethically approved by the Dutch Committee of Research Involving Human Subjects (CCMO; number NL38237.042.11). Parental written informed consent was obtained at T1. The TRAILS(-cc) participants also gave written informed consent from the second assessment wave onwards.

### Measures

#### Main variables

##### Autistic-like features

Autistic-like features were assessed at T1 through T4 using the Children’s Social Behavior Questionnaire (CSBQ) (Hartman et al., 2006). The CSBQ was developed to assess behaviors that represent core symptoms of ASD, also including more subtle variants of these behaviors that often occur at the milder end of the spectrum. It contains 49 items divided into six scales: “not optimally tuned to the social situation” (tuned; 11 items), “reduced contact and social interest” (social; 12 items), “orientation problems in time, place, or activity” (orientation; 8 items), “difficulties in understanding of social information” (understanding; 7 items), “fear of and resistance to changes” (change; 3 items), and “stereotyped behavior” (stereotyped; 8 items). Psychometric properties were good (de Bildt et al., 2009; Hartman et al., 2006). The parents of the TRAILS(-cc) participants indicated to which extend the stated behavior had applied to their child during the preceding two months, by rating each item on a three-point Likert-type scale (0 = does not apply, 1 = sometimes or somewhat applies, 2 = clearly or often applies). Internal consistency in our study was excellent (T1 α = 0.94; T2 α = 0.95; T3 α = 0.95; T4 α = 0.96).

##### FSS

FSS were measured using the Somatic Complaints subscale of the Youth Self Report (YSR) (Achenbach & Rescorla, 2003) at T1 through T3. At T4, the Adult Self Report (ASR) (Achenbach & Rescorla, 2001) was used, suiting the age of the participants at that time. The Somatic Complaints subscale contains items that refer to somatic complaints without a known medical cause or without obvious reason. The ASR contains three more items than the YSR, which are heart pounding, numbness, and trouble sleeping. These items were excluded from analyses to ensure consistency between the two versions. Another two items, skin problems and eye problems, were excluded from analyses due to low factor loadings reported in previous TRAILS studies (Janssens et al., 2010, 2014). The remaining seven items were: aches/pains, headache, nausea, stomach pain, vomiting, overtiredness, and dizziness. The TRAILS(-cc) participant indicated to which extent the stated complaint had applied to him or her during the past 6 months, by rating each item on a three-point Likert-type scale (0 = never or not at all true, 1 = sometimes or a bit true, 2 = often or very true). Internal consistency of the seven items used in our study was good (T1 α = 0.75; T2 α = 0.77; T3 α = 0.75; T4 α = 0.78).

#### Secondary variables

##### Sex

Sex was assessed at T1 using self-report. It was coded as female: 0 or male: 1.

##### Psychotropic medication

The use of psychotropic medication was included as a covariate. It was assessed using parent report at T1 through T3 and parent report and self-report at T4. At T4, use was coded as present when either parent or adolescent answered affirmatively. Five types of medication were included: antipsychotics, anxiolytics, sedatives and hypnotics, anti-depressants, and stimulants. Medication was classified using the Anatomical Therapeutic Chemical Classification System (World Health Organization, 2004). Psychotropic medication use was used as a single category (coded as present: 1 or absent: 0).

##### Socioeconomic status

Socioeconomic status (SES) was measured at T1 using the occupational level and educational level of the father and the mother, and average household income as indicators. The indicator occupation was transformed to a Z-score, based on the International Standard Classification of Occupations (Ganzeboom & Treiman, 1996). The other indicators (educational level and household income) were also transformed to Z-scores. The Z-scores of all indicators were then summed up and transformed to a new Z-score.

##### Chronic disease

The presence of the chronic diseases asthma and diabetes, the two most common chronic diseases among adolescents (Dabelea et al., 2014; Dharmage et al., 2019), was assessed at T1 through T3 using parent report. It was coded as present: 1 or absent: 0.

##### ASD diagnosis

Clinical ASD diagnoses were retrieved from the Psychiatric Case Register Northern Netherlands (PCRNN). The PCRNN contains specialist mental healthcare utilization in the northern Netherlands from 2000 onwards. For TRAILS-cc, these data were enriched with diagnoses retrieved at the psychiatric outpatient clinic from the child’s healthcare files up to age 11. If an ASD diagnosis was listed in either the PCRNN or healthcare file, it was coded as present: 1. All other cases were coded as absent: 0.

### Statistical analyses

This study was preregistered prior to analysis of the data (https://osf.io/3g5yq). As described herein, first, descriptive statistics were calculated using SPSS version 26. Subsequently, we used the random intercept cross-lagged panel model (RI-CLPM) in Mplus to investigate the prospective interrelations between autistic-like features and FSS. The RI-CLPM is an extension of the traditional cross-lagged panel model and incorporates autoregressive and cross-lagged parameters as well as random intercepts (Hamaker et al., 2015). The RI-CLPM differentiates between a stable time-invariant between-persons part, and a fluctuating part at a within-person level. The latter part of the model captures the direct effects from autistic-like features at one time point to FSS at a subsequent time point and vice versa, and may thus provide insight into potentially causal pathways present during the developmental period that is studied. The stable, time-invariant part of the model shows the stable correlations between autistic-like features and FSS during the period of study. In addition, the RI-CLPM estimates the autoregressive effects and wave-specific residual associations between autistic-like features and FSS.

First, intraclass correlations (ICCs) were calculated for item mean scores of autistic-like features and FSS. Intraclass correlations reveal to what extent autistic-like features are correlated across all waves, and likewise, to what extent FSS are correlated across all waves. This intraclass correlation represents the proportion of (stable) between-persons variance, the remaining represents the variance explained by fluctuations over time within persons. Next, the RI-CLPM model was fitted to the data. SES and sex as time-invariant variables and psychotropic medication use as time-varying variable were used as covariates in the model. SES was included as covariate, since low SES is a known risk factor for both somatic and psychosocial problems (Bradley & Corwyn, 2002; Hinz et al., 2017). Sex was included because ASD is more prevalent in males, while FSS are more prevalent in females (Beck, 2008; Werling & Geschwind, 2013). Also, symptoms of both could present differently in males than females (Barsky et al., 2001; Werling & Geschwind, 2013). This possibly leads to different associations. Psychotropic medication may be prescribed in autistic children (mostly for comorbid problems) and may produce somatic side effects (Garcia et al., 2012), therefore possibly influencing the relation between autistic-like features and FSS.

Subsequently, different RI-CLPMs were run separately for autistic-like social and communication behaviors (CSBQ subscales “reduced contact and social interests” and “difficulties in understanding social information”), for autistic-like repetitive behaviors (CSBQ subscales “stereotyped behavior” and “fear of and resistance to changes”), and for autistic-like self-regulatory behaviors (CSBQ subscales “not optimally tuned to the social situation” and “orientation problems in time, place, or activity”) to explore whether the association between autistic-like features and FSS differs per type of autistic-like features. Next, a sensitivity analysis was performed in which we excluded all participants with asthma or diabetes, to test if the presence of a chronic disease affected the results. Another sensitivity analysis was performed in which cohort status (TRAILS or TRAILS-cc) was added as a covariate to test whether differences between the cohorts in demographic characteristics affected the results. Last, the RI-CLPM used for the main analysis was rerun in the subsample of TRAILS and TRAILS-cc participants with a clinical ASD diagnosis, to explore whether the interrelations between autistic-like features and FSS were different in these adolescents.

Adequate model fit was indicated by the following values: root mean squared error approximation (RMSEA) smaller than 0.05, standardized root mean squared residual (SRMR) smaller than 0.08, and comparative fit index (CFI) greater than 0.95 (Hu & Bentler, 1999).

Respondents with missing data on autistic-like features and/or FSS on two or less waves were included in the analyses. We handled these missing data by using full information maximum likelihood (FIML) estimation (Little & Rubin, 2002). Participants with missing data on autistic-like features and/or FSS on three or more assessment waves were excluded from the analyses. All analyses were performed in Mplus version 8 (Muthén & Muthén, 1998–2017).

### Community involvement

There was no community involvement in the reported study.

## Results

### Descriptive findings

Table 1 shows the descriptive statistics of the variables included in the model (descriptives split by cohort can be found in Table S1, available online). Autistic-like features show high stability, although a slight decrease over time seems to be apparent. FSS appear to decrease over time to a larger extent. The percentage of psychotropic medication users first increases at T2 and T3 compared to T1, and then decreases at T4.

**Table 1.** Descriptives.

Variable	T1 (*n* = 2772)	T2 (*n* = 2610)	T3 (*n* = 2237)	T4 (*n* = 2302)
Clinical––cohort *n* (%)^a^	543 (19.6)	462 (17.7)	419 (18.7)	422 (18.3)
Sex—males *n* (%)^a^	1456 (52.5)	1360 (52.1)	1144 (51.1)	1176 (51.1)
Age—years *M* (*SD)*	11.11 (0.55)	13.44 (0.61)	16.21 (0.72)	19.09 (0.63)
Autistic-like features—*M* (*SD*)^b^	0.33 (0.29)	0.28 (0.28)	0.27 (0.28)	0.25 (0.28)
FSS—*M* (*SD*)^c^	0.47 (0.35)	0.39 (0.35)	0.34 (0.34)	0.22 (0.31)
Psychotropic medication use—*n* (%)^a^	297 (11.6)	335 (14.1)	241 (12.5)	200 (8.9)
Chronic diseases—*n* (%)^a^	202 (7.3)	156 (6.0)	111 (5.0)	n/a
Socioeconomic status—*M* (*SD*)	−0.05 (0.79)	n/a	n/a	n/a

Note. FSS: functional somatic symptoms; CSBQ: Children’s Social Behavior Questionnaire; YSR: Youth Self Report; ASR: Adult Self Report.

a Percentage based on total sample without missing data.

b Item mean score of the CSBQ, theoretical range 0–2.

c Item mean score of seven included items of the Somatic Complaints scale of the YSR (T1 T3) or ASR (T4), theoretical range 0–2.

### Stability in autistic-like features and FSS across the waves

Based on intraclass correlation, 71.3% of the variance in autistic-like features across the four assessment waves was explained by stable between-person differences. The remaining 28.7% was explained by within-person fluctuations at the different assessment waves. For FSS, 28.0% of the variance was explained by between-person differences, and the remaining 72.0% by within-person fluctuations.

### Developmental interrelations between autistic-like features and FSS

The RI-CLPM was run with all participants of whom data on autistic-like features and FSS were available on two or more waves (*n* = 2407). We had initially planned to include sex as covariate. The model did, however, not converge when doing so. Therefore, deviating from the preregistration, multi-group analyses of males and females were performed. The model with freely estimated parameters achieved good fit (χ^2^ (86) = 200.61, *p* < 0.01 CFI = 0.979, RMSEA = 0.033, SRMR = 0.029). Subsequently, as shown in Table 2, similarities between males and females were tested by adding equality constraints to estimates across the sexes in a blockwise manner (blocks by order: covariates, stable correlations, within-wave (residual) correlations, cross-lagged paths, autoregressive paths autistic-like features, autoregressive paths FSS). Model fit deterioration was evaluated using Satorra-Bentler scaled χ^2^-difference tests (Satorra & Bentler, 2001), comparing nested models to the model with freely estimated parameters. If two models fitted the data equally well, the most parsimonious model was chosen. This would indicate similar effects for males and females. If the model fit deteriorated significantly, indicating different effects for males and females, the equality constraints were relaxed again. The final estimated model indicated that males and females did not differ with respect to stable correlations (random intercepts), within-wave (residual) correlations, cross-lagged paths, and autoregressive paths of autistic-like features. Effects of covariates (SES and psychotropic medication) and autoregressive paths of FSS differed for males and females. Regarding the effect of covariates, the relation between psychotropic medication and autistic-like features at T4 was significant for males but not for females; the relation between psychotropic medication and FSS at T1 was significant for males but not for females; there were no differences between males and females in the effects of SES. Regarding the autoregressive effects, the path between FSS at T3 and FSS at T4 was significant for females but not for males. The optimal (constrained) model achieved good fit (χ^2^ (100) = 211.997, *p* < 0.01, CFI = 0.979, RMSEA = 0.031, SRMR = 0.031).

**Table 2.** Fit statistics for model comparisons in multi-group analyses.

Model	χ^2^ (df)	CFI	RMSEA	SRMR	Model comparison (compared with model 1): χ^2^ (df), *p*
1—Freely estimated	200.614 (86)	0.979	0.033	0.029	n/a
2—Constraints on covariates	231.013 (96)	0.975	0.034	0.040	30.217 (10) *p* = 0.001*
3—Constraints on stable correlations (random intercepts)	200.495 (87)	0.979	0.033	0.029	0.122 (1) *p* = 0.727
4—Constraints on stable correlations and within-wave correlations	206.094 (91)	0.978	0.032	0.030	5.333 (5) *p* = 0.377
5—Constraints on stable correlations, within-wave correlations, and cross-lagged paths	211.955 (97)	0.978	0.031	0.031	10.967 (11) *p* = 0.446
6—Constraints on stable correlations, within-wave correlations, cross-lagged paths, and autoregressive paths autistic-like features^a^	211.997 (100)	0.979	0.031	0.031	13.785 (14) *p* = 0.466
7—Constraints on stable correlations, within-wave correlations, cross-lagged paths, and autoregressive paths autistic-like features and FSS	232.181 (103)	0.976	0.032	0.034	32.265 (17) *p* = 0.014*

CFI: comparative fit index; RMSEA: root mean squared error approximation; SRMR: standardized root mean squared residual; FSS: functional somatic symptoms.

a Optimal constrained model.

* Significant at p < 0.05.

**Figure 2.**
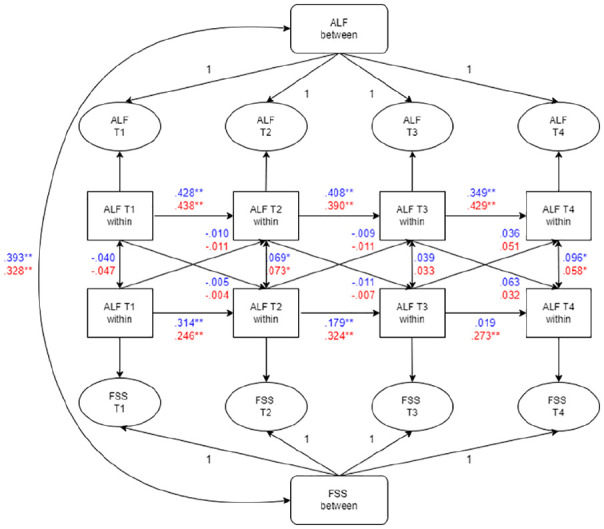
Summary of multi-group analyses for males and females in the optimal constrained RI-CLPM. Coefficients in blue = male; coefficients in red = female; ALF = autistic-like features; RI-CLPM: random intercept cross-lagged panel model. *Significant at *p* < 0.05; **significant *p* < 0.01.

As Figure 2 shows, a positive, moderately strong association between autistic-like features and FSS was found at the between-person level (*b* = .008, after sex-specific standardization: male: β = 0.393, *p* < 0.01; female: β = 0.328, *p* < 0.01). This indicates that adolescents with higher levels of autistic-like features across the four waves also reported higher levels of FSS across the four waves. None of the cross-lagged relations was significant, meaning that we found no evidence for reciprocal associations between autistic-like features and FSS over the repeated measures. In other words, in our sample, changes in autistic-like features were unrelated to changes in FSS at the subsequent wave, and vice versa, changes in FSS were unrelated to changes in autistic-like features at the subsequent wave. More detailed information on the parameter estimates can be found in Supplementary Text 1 (available online).

To explore whether the association between autistic-like features and FSS differed per type of autistic-like features, three RI-CLPMs were run separately for three domains of autistic-like features. The results show that the parameter estimates of all three models were similar to those of the model with the total of autistic-like features. Tables S2–S4 (available online) show the fit statistics. Figures S1–S3 (available online) visualize the models and results.

To test in sensitivity analyses if the presence of chronic diseases influenced our results, we reran the complete model (including the total of autistic-like features) while excluding all participants with asthma or diabetes on at least one of the assessment waves (*n* = 247). Fit statistics can be found in Table S5 (available online). Figure S4 (available online) summarizes the results. The results were similar with respect to stable association and cross-lagged paths. More detailed information on the parameter estimates can be found in Supplementary Text 2 (available online).

To test in sensitivity analyses if meaningful differences between the cohorts in demographic characteristics influenced our results, we reran the complete model while adding cohort status as a covariate. Fit statistics can be found in Table S6 (available online). Figure S5 (available online) summarizes the results. The results were highly similar to the main analyses with respect to all parameter estimates.

To explore whether the interrelations between autistic-like features and FSS were different for adolescents diagnosed with ASD, we reran the complete model in a subsample of participants with a clinical ASD diagnosis (*n* = 264; 75.8% male). Fit statistics can be found in Table S7 (available online). Figure S6 (available online) summarizes the results. Similar to the analysis performed in the total sample, a positive association between autistic-like features and FSS was found at the between-person level and none of the cross-lagged relations were significant. More detailed information on the parameter estimates can be found in Supplementary Text 3 (available online).

## Discussion

The current study examined the longitudinal relations between autistic-like features and FSS during adolescence. We found a stable positive moderate between-person association between autistic-like features and FSS over time. Beyond this stable association, we found no reciprocal effects from wave to wave between autistic-like features and FSS. In other words, contrary to our hypothesis, we found no evidence that a change in autistic-like features contributed to a subsequent change in FSS, nor that a change in FSS contributed to a subsequent change in autistic-like features. Rather, in our sample, the between-person association between autistic-like features and FSS was stable over the course of adolescence in males and females. The relation with FSS was consistent for all different domains of autistic-like features.

This study has several strengths. The four assessment waves covered the complete developmental period of adolescence. This allowed us to study associations over this entire period, which is an important time in terms of social and physical development, and thus, highly relevant for processes of autistic-like features and FSS. In addition, we used a model which enabled us to distinguish between stable between-person associations and fluctuating within-person associations. Other strengths are the use of well-validated instruments to assess the key variables, and the use of parent reported autistic-like features and self- reported FSS symptoms. Parents and adolescents perceive symptoms differently. Due to the nature of the symptoms, parents are considered more appropriate informants for autistic-like features (Johnson et al., 2009), whereas self-report is more appropriate for measuring FSS (van de Looij-Jansen et al., 2011). Possibly, we would have found stronger relations when the same informant was used for measuring autistic-like features and FSS. Using different informants reduced the risk of inflating the stable relation between autistic-like features and FSS by single-informant bias. Last, by using data from both the population cohort and clinical cohort, we were able to investigate associations in a sample that included adolescents with lower as well as higher symptomatology, thus ensuring adequate (co-)variance. Our sensitivity analysis yielded highly similar results when cohort status was included as covariate, indicating that differences between the cohorts in demographic or other potentially confounding characteristics did not affect our results. In addition, we found similar interrelations in the subsample of participants with a clinical ASD diagnosis as in the total sample. This indicates that also in adolescents with autistic-like features above the clinical certain threshold, reciprocal relations are unlikely to be present.

A few limitations should be taken into account when interpreting the results. First, as in any longitudinal study, we had to deal with attrition, which was not random. Participants with missing data on three or more waves were not included in the analyses. Since participants with more severe symptomatology were more likely to drop out (Oldehinkel et al., 2015), this could have led to an underestimation of associations between autistic-like features and FSS. Although we handled missing data using FIML, attrition may have biased the findings. Second, we cannot exclude the possibility that certain somatic symptoms were part of a physical disease. Although the YSR and ASR specifically assess symptoms that cannot be explained by a medical cause, there is always a possibility that somatic symptoms are caused by a disease that has not been detected yet. However, for the processes that we studied, it may not matter whether the symptoms were medically explained or not. Mechanisms that influence symptom experience, such as heightened attention to sensory stimuli, play a role in all somatic symptoms, regardless of the cause (Crombez et al., 2005). Accordingly, our sensitivity analysis showed that excluding participants with asthma or diabetes, the most common chronic diseases among adolescents (Dabelea et al., 2014; Dharmage et al., 2019), did not change the results. Third, we assessed autistic-like features using the CSBQ, which partly measures in two subscales autistic-like features outside the core features of ASD. That is, the subscales in the domain “self-regulatory behaviors” measure symptoms that are characteristic of ASD but not specific for ASD as they also capture behaviors seen in children with attention deficit hyperactivity disorder and oppositional defiant disorder (de Bildt et al., 2009; Hartman et al., 2006). However, our secondary analyses, which made the distinction between the two core domains of ASD (i.e. social and communication behaviors and repetitive behaviors) and the self-regulatory domain, showed similar relations with FSS for all three problem domains.

We are the first to study reciprocal associations between autistic-like features and FSS during adolescence using a longitudinal design. The stable association that we found between autistic-like features and FSS is an extension of previous cross-sectional studies that showed elevated FSS in autistic adolescents compared to non-autistic peers (Paul et al., 2015; Schroeder et al., 2011). It is also in line with the finding that school-aged children with more severe autistic-like features exhibit higher levels of FSS compared to those with autistic-like features of a lower severity (Kim et al., 2020). Longitudinal studies on this topic have so far been scarce. One study examined longitudinal relations between autistic-like features and internalizing traits across middle to late childhood, and, contrary to ours, did find reciprocal effects from wave to wave (Hallett et al., 2010). That study differs from ours in terms of the developmental period that was studied, and the use of an aggregate of internalizing traits, which included only one item assessing somatic symptoms, next to items concerning anxiety and depression. Importantly, unlike the RI-CLPM that we used in the current study, the model that this previous study used did not disentangle stable interpersonal associations and fluctuating intrapersonal associations. Longitudinal associations may thus emerge that do not reflect developmental changes but are rather part of the overall stability in how different problem domains are related. Our model avoided these pitfalls, which could explain the different findings in our study.

Our results show that the relation between autistic-like features and FSS is rather stable during adolescence. Earlier proposed mechanisms explained how reciprocal influences might take effect in different types of autistic-like features. However, our findings did not show differences in the stable relation between autistic-like features and FSS for different autistic-like feature domains, nor did they provide any support for a reciprocal influence between autistic-like features and FSS during adolescence. Reciprocal effects possibly occur earlier in childhood, before remaining stable throughout adolescence. Alternatively, the presence of other variables and/or a more complex relation can explain the co-occurrence. Possibly, psychological concepts closely related to autistic-like features can provide part of an explanation. As such, a recent study found that alexithymia (difficulty in identifying and describing own emotions) and intolerance of uncertainty were predictive of somatic symptoms in both autistic and non-autistic adults, regardless of autism status (Larkin et al., 2022). It is, however, very difficult to disentangle these two predictors from autistic-like features. Both are elevated in autistic individuals and show relations to (severity of) core features of ASD (Jenkinson et al., 2020; Kinnaird et al., 2019; Vasa et al., 2018). Other mental health problems may be a shared factor contributing to the stable association that we found. Indeed, previous TRAILS studies found associations between mental health problems and both autistic-like features and FSS, respectively (Horwitz et al., 2020; Janssens et al., 2010). A recent study showed that in autistic young adults, somatic symptom burden was, next to higher levels of autistic-like features, associated with more severe symptoms of anxiety and depression. However, autistic-like features remained significantly predictive of somatic symptoms after accounting for co-occurring psychopathology, indicating that autistic-like features form an independent risk factor (Williams & Gotham, 2022). Stressful life events, may elicit, perpetuate or exacerbate both autistic-like features and FSS (Bonvanie et al., 2017; Taylor & Gotham, 2016). Previous research has also shown associations between autism and somatic health conditions (Muskens et al., 2017). In the current study, we specifically focused on associations between autistic-like features and FSS, and therefore, we did not include other mental or somatic health conditions. Future research may focus on associations with other conditions to refine this knowledge.

We also found that autistic-like features are rather stable over time and FSS to a far lesser extent, as was also indicated by the ICCs (ASD: 71.3%; FSS: 28.0%). It could be argued that the comparatively high stability in autistic-like features makes fluctuations as a function of FSS unlikely. Within-person fluctuations in autistic-like features are, however, not uncommon in adolescents (Bal et al., 2019). Most of the autistic participants were on the milder side of the autism spectrum, in whom individual differences in developmental change are to be expected (Louwerse et al., 2015). Possibly, reciprocal effects between autistic-like features and FSS do occur but within shorter time intervals. We could not capture those with our bi-annual assessment waves. Our main interest, however, was in longer-term developmental pathways.

What causes autistic-like features and FSS to co-occur is still largely unknown. Future research could focus on other variables that could explain the co-occurrence. These might be factors like shared genetic background or mental health problems. Studying interrelations in young children could shed light on potential reciprocal effects at a younger age. This would also be interesting because certain FSS, including abdominal pain, decrease from childhood into adolescence (Janssens et al., 2011). Moreover, by starting to measure at an early age, possibly even before symptoms become apparent, processes regarding the emergence of (co-occurring) symptoms may become clearer. Indeed, the co-occurrence between autistic-like features and FSS may have already been established before adolescence. Further insight into potential mechanisms and variables that explain the co-occurrence of autistic-like features and somatic symptoms will be useful to improve preventive and treatment strategies for co-occurring symptoms. For now, we have shown stable moderate associations between autistic-like features and FSS between late childhood and young adulthood that are unbiased by single-informant bias. The results of this study may alert clinicians to co-existing autistic-like features and FSS. Adolescents presenting with co-occurring symptoms might benefit from tailored interventions. Treatment focusing on FSS may be adapted to better suit adolescents with autistic-like features. In turn, more attention could be paid to FSS in treatment focusing on autistic-like features.

## Supplemental Material

sj-docx-1-aut-10.1177_13623613221143874 – Supplemental material for Longitudinal relations between autistic-like features and functional somatic symptoms in adolescence Click here for additional data file. Supplemental material, sj-docx-1-aut-10.1177_13623613221143874 for Longitudinal relations between autistic-like features and functional somatic symptoms in adolescence by Elske Hogendoorn, Catharina A Hartman, Sarah M Burke, Marijn W G van Dijk and Judith G M Rosmalen in Autism
