# Supplementary material for: Longitudinal relations between autistic-like features and functional somatic symptoms in adolescence
Source: Autism. 2023 Jan 1;27(6):1690–701. doi: 10.1177/13623613221143874 (PMC10375000; doi:10.1177/13623613221143874)
Supplement: sj-docx-1-aut-10.1177_13623613221143874 – Supplemental material for Longitudinal relations between autistic-like features and functional somatic symptoms in adolescence [file sj-docx-1-aut-10.1177_13623613221143874.docx]

| **Cohort** | **Variable** | **T1 (n=2772)** | **T2 (n=2610)** | **T3 (n=2237)** | **T4 (n=2302)** |
| --- | --- | --- | --- | --- | --- |
| TRAILS | n | 2229 | 2148 | 1818 | 1880 |
|  | Sex – Males *n* (%)*^a^* | 1098 (49.3) | 1054 (49.1) | 867 (47.7) | 898 (47.8) |
|  | Age – Years *M (SD)* | 11.11 (0.56) | 13.57 (0.53) | 16.28 (0.71) | 19.08 (0.60) |
|  | Autistic-like features – *M (SD)^b^* | 0.26 (0.23) | 0.21 (0.21) | 0.19 (0.21) | 0.20 (0.24) |
|  | FSS – *M (SD)*^c^ | 0.46 (0.35) | 0.39 (0.35) | 0.34 (0.34) | 0.20 (0.31) |
|  | Psychotropic medication use – *n (%)^a^* | 45 (2.2) | 109 (5.7) | 62 (4.1) | 90 (4.9) |
|  | Chronic diseases – *n (%)^a^* | 136 (6.1) | 110 (5.1) | 79 (4.3) | n/a |
|  | Socioeconomic status – *M (SD)* | -0.05 (0.80) | n/a | n/a | n/a |
| TRAILS-cc | n | 543 | 462 | 419 | 422 |
|  | Sex – Males *n* (%)*^a^* | 358 (65.9) | 306 (66.2) | 277 (66.1) | 278 (65.9) |
|  | Age – Years *M (SD)* | 11.11 (0.50) | 12.86 (0.62) | 15.91 (0.66) | 19.11 (0.73) |
|  | Autistic-like features – *M (SD)^b^* | 0.60 (0.34) | 0.57 (0.35) | 0.53 (0.35) | 0.46 (0.34) |
|  | FSS – *M (SD)*^c^ | 0.47 (0.35) | 0.42 (0.36) | 0.34 (0.34) | 0.30 (0.33) |
|  | Psychotropic medication use – *n (%)^a^* | 252 (46.4) | 226 (49.9) | 179 (43.2) | 110 (27.2) |
|  | Chronic diseases – *n (%)^a^* | 66 (12.2) | 46 (10.0) | 32 (7.6) | n/a |
|  | Socioeconomic status – *M (SD)* | -0.05 (0.74) | n/a | n/a | n/a |

**Table S1.** Descriptives split by cohort.

Note. a = percentage based on total sample without missing data; b = item mean score of the CSBQ, range 0-2; c = item mean score of seven included items of the Somatic Complaints scale of the YSR (T1-T3) or ASR (T4), theoretical range 0-2.

**Table S2.** Fit statistics for model comparisons in multi-group analyses for social and communication behaviors and FSS.

| **Model** | **χ^2^ (df)** | **CFI** | **RMSEA** | **SRMR** | **Model comparison (compared with model 1): χ^2^ (df), *p*** |
| --- | --- | --- | --- | --- | --- |
| Model 1 – Freely estimated | 478.751 (88) | 0.939 | 0.061 | 0.057 | n/a |
| Model 3 – Constraints on covariates | 491.052 (98) | 0.939 | 0.058 | 0.061 | 16.068 (10)  *p* = 0.098 |
| Model 4 – Constraints on covariates and stable correlations (random intercepts) | 490.290 (99) | 0.939 | 0.057 | 0.061 | 16.292 (11)  *p* = 0.131 |
| Model 5 – Constraints on covariates, stable correlations and within-wave correlations | 501.609 (103) | 0.938 | 0.057 | 0.061 | 26.784 (15)  *p* = 0.031* |
| Model 6 – Constraints on covariates, stable correlations, and cross-lagged paths | 504.413 (105) | 0.938 | 0.056 | 0.062 | 29.257 (17)  *p* = 0.032* |
| Model 7 ** – Constraints on covariates, stable correlations, and autoregressive paths social and communication behaviors | 428.897 (102) | 0.941 | 0.056 | 0.061 | 15.400 (14)  *p* = 0.351 |
| Model 8 – Constraints on covariates, stable correlations, and autoregressive paths social and communication behaviors and FSS | 504.451 (105) | 0.938 | 0.056 | 0.062 | 33.970 (17)  *p* = 0.008* |

Note. * = significant at p < 0.05, ** = optimal constrained model

**Table S3.** Fit statistics for model comparisons in multi-group analyses for repetitive behaviors and FSS.

| **Model** | **χ^2^ (df)** | | **CFI** | **RMSEA** | **SRMR** | **Model comparison (compared with model 1): χ^2^ (df), *p*** |
| --- | --- | --- | --- | --- | --- | --- |
| Model 1 – Freely estimated | 214.020 (88) | 0.965 | | 0.034 | 0.031 | n/a |
| Model 2 – Constraints on covariates | 243.755 (98) | 0.959 | | 0.035 | 0.042 | 29.416 (10)  *p* = 0.001* |
| Model 3 – Constraints on stable correlations (random intercepts) | 214.724 (89) | 0.965 | | 0.034 | 0.031 | 0.914 (1)  *p* = 0.339 |
| Model 4 – Constraints on stable correlations and within-wave correlations | 216.695 (93) | 0.965 | | 0.033 | 0.032 | 2.893 (5)  *p* = 0.716 |
| Model 5 – Constraints on stable correlations, within-wave correlations, and cross-lagged paths | 238.283 (99) | 0.961 | | 0.034 | 0.034 | 24.205 (11)  *p* = 0.012* |
| Model 6 ** – Constraints on stable correlations, within-wave correlations, and autoregressive paths repetitive behaviors and FSS | 216.002 (99) | 0.967 | | 0.031 | 0.033 | 7.360 (11)  *p* = 0.769 |

Note. * = significant at p < 0.05, ** = optimal constrained model

**Table S4.** Fit statistics for model comparisons in multi-group analyses for self-regulatory behaviors and FSS.

| **Model** | **χ^2^ (df)** | **CFI** | **RMSEA** | **SRMR** | **Model comparison (compared with model 1): χ^2^ (df), *p*** |
| --- | --- | --- | --- | --- | --- |
| Model 1 – Freely estimated | 222.060 (88) | 0.975 | 0.036 | 0.032 | n/a |
| Model 2 – Constraints on covariates | 250.145 (98) | 0.972 | 0.036 | 0.041 | 26.171 (10)  *p* = 0.004* |
| Model 3 – Constraints on stable correlations (random intercepts) | 222.565 (89) | 0.975 | 0.035 | 0.032 | 0.662 (1)  *p* = 0.416 |
| Model 4 – Constraints on stable correlations and within-wave correlations | 228.264 (93) | 0.975 | 0.035 | 0.033 | 6.231 (5)  *p* = 0.284 |
| Model 5 – Constraints on stable correlations, within-wave correlations, and cross-lagged paths | 231.958 (99) | 0.975 | 0.033 | 0.033 | 9.354 (11)  *p* = 0.589 |
| Model 6 ** – Constraints on stable correlations, within-wave correlations, cross-lagged paths, and autoregressive paths self-regulatory behaviors | 233.693 (102) | 0.976 | 0.033 | 0.033 | 13.834 (14)  *p* = 0.463 |
| Model 7 – Constraints on stable correlations, within-wave correlations, cross-lagged paths, and autoregressive paths self-regulatory behaviors and FSS | 55.807 (115) | 0.972 | 0.035 | 0.036 | 34.392 (17)  *p* = 0.007* |

Note. * = significant at p < 0.05, ** = optimal constrained model

**Table S5.** Fit statistics for model comparisons in multi-group sensitivity analyses in which all participants with chronic diseases were excluded.

| **Model** | **χ^2^ (df)** | **CFI** | **RMSEA** | **SRMR** | **Model comparison (compared with model 1): χ^2^ (df), *p*** |
| --- | --- | --- | --- | --- | --- |
| Model 1 – Freely estimated | 189.937  (86) | 0.978 | 0.033 | 0.030 | n/a |
| Model 2 – Constraints on covariates | 218.816 (96) | 0.974 | 0.034 | 0.040 | 28.661 (10)  *p* = 0.001* |
| Model 3 – Constraints on stable correlations (random intercepts) | 189.661 (87) | 0.979 | 0.033 | 0.030 | 0.013 (1)  *p* = 0.910 |
| Model 4 – Constraints on stable correlations and within-wave correlations | 195.625 (91) | 0.978 | 0.033 | 0.031 | 5.590 (5)  *p* = 0.348 |
| Model 5 – Constraints on stable correlations, within-wave correlations, and cross-lagged paths | 201.485 (97) | 0.978 | 0.032 | 0.032 | 11.426 (11)  *p* = 0.408 |
| Model 6 – Constraints on stable correlations, within-wave correlations, cross-lagged paths, and autoregressive paths | 221.046 (103) | 0.975 | 0.033 | 0.035 | 31.835 (17)  *p* = 0.016* |
| Model 7** – Constraints on stable correlations, within-wave correlations, cross-lagged paths, and autoregressive paths autistic-like features | 200.064 (100) | 0.979 | 0.030 | 0.032 | 13.025 (14)  *p* = 0.525 |
| Model 8 – Constraints on stable correlations, within-wave correlations, cross-lagged paths, and autoregressive paths autistic-like features and FSS | 221.046 (103) | 0.975 | 0.033 | 0.035 | 31.835 (17)  *p* = 0,016* |

Note. * = significant at p < 0.05, ** = optimal constrained model

**Table S6.** Fit statistics for model comparisons in multi-group sensitivity analyses in which cohort was added as covariate.

| **Model** | **χ^2^ (df)** | **CFI** | **RMSEA** | **SRMR** | **Model comparison (compared with model 1): χ^2^ (df), *p*** |
| --- | --- | --- | --- | --- | --- |
| Model 1 – Freely estimated | 987.245 (116) | 0.842 | 0.078 | 0.107 | n/a |
| Model 2 – Constraints on covariates | 991.271 (120) | 0.842 | 0.078 | 0.107 | 2.231 (4)  *p* = 0.693 |
| Model 3 – Constraints on covariates and stable correlations (random intercepts) | 990.472 (121) | 0.842 | 0.077 | 0.107 | 2.390 (5)  *p* = 0.793 |
| Model 4 – Constraints on covariates, stable correlations and within-wave correlations | 998.120 (125) | 0.841 | 0.076 | 0.107 | 7.534 (9)  *p* = 0.582 |
| Model 5 – Constraints on covariates, stable correlations, within-wave correlations, and cross-lagged paths | 1006.679 (131) | 0.841 | 0.075 | 0.107 | 12.378 (15)  *p* = 0.650 |
| Model 6 **– Constraints on covariates, stable correlations, within-wave correlations, cross-lagged paths, and autoregressive paths autistic-like features | 995.171 (134) | 0.843 | 0.073 | 0.107 | 15.120 (18)  *p* = 0.654 |
| Model 7 – Constraints on covariates, stable correlations, within-wave correlations, cross-lagged paths, and autoregressive paths autistic-like features and FSS | 1016.767 (137) | 0.840 | 0.073 | 0.108 | 34.210 (21)  *p* = 0.034* |

Note. * = significant at p < 0.05, ** = optimal constrained model

**Table S7.** Fit statistics for model comparisons in multi-group analyses in a subsample of participants with a clinical ASD diagnosis.

| **Model** | **χ^2^ (df)** | **CFI** | **RMSEA** | **SRMR** | **Model comparison (compared with model 1): χ^2^ (df), *p*** |
| --- | --- | --- | --- | --- | --- |
| Model 1 – Freely estimated | 85.805 (86) | 1.000 | 0.000 | 0.048 | n/a |
| Model 2 – Constraints on covariates | 95.218 (96) | 1.000 | 0.000 | 0.057 | 9.444 (10)  *p* = 0.491 |
| Model 3 – Constraints on covariates and stable correlations (random intercepts) | 95.631 (97) | 1.000 | 0.000 | 0.056 | 9.884 (11)  *p* = 0.541 |
| Model 4 – Constraints on covariates, stable correlations and within-wave correlations | 97.627 (101) | 1.000 | 0.000 | 0.059 | 11.906 (15)  *p* = 0.686 |
| Model 5 – Constraints on covariates, stable correlations, within-wave correlations, and cross-lagged paths | 106.094 (107) | 1.000 | 0.000 | 0.058 | 20.306 (21)  *p* = 0.502 |
| Model 6 – Constraints on covariates, stable correlations, within-wave correlations, cross-lagged paths, and autoregressive paths autistic-like features | 110.813 (110) | 0.999 | 0.007 | 0.060 | 24.973 (24)  *p* = 0.407 |
| Model 7** – Constraints on covariates, stable correlations, within-wave correlations, cross-lagged paths, and autoregressive paths autistic-like features and FSS | 117.381 (113) | 0.993 | 0.017 | 0.063 | 31.315 (27)  *p* = 0.258 |

Note. ** = optimal constrained model

**Figure S1**. Summary of multi-group analyses in the optimal constrained RI-CLPM for social and communication behaviors and FSS.


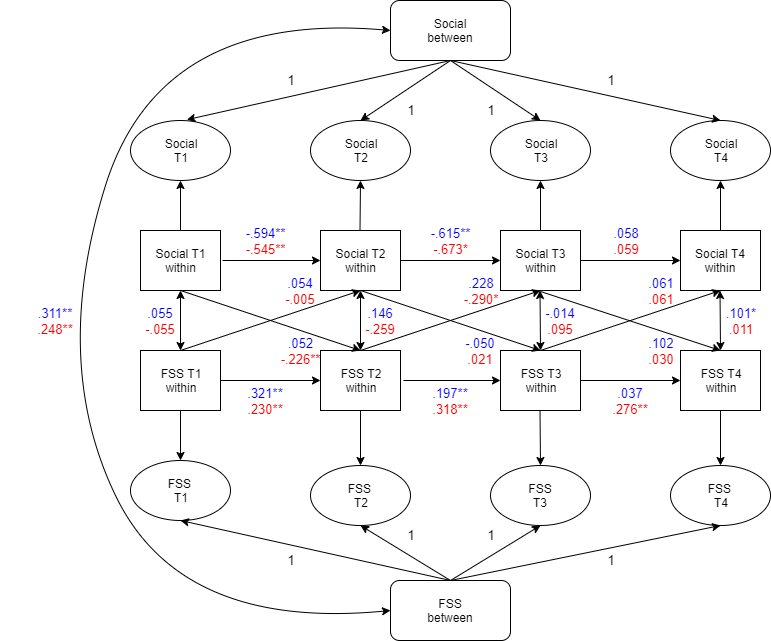


Note. * = significant at p < 0.05; ** = significant p < 0.01; coefficients in blue = male; coefficients in red = female.

**Figure S2**. Summary of multi-group analyses in the optimal constrained RI-CLPM for repetitive behaviors and FSS.


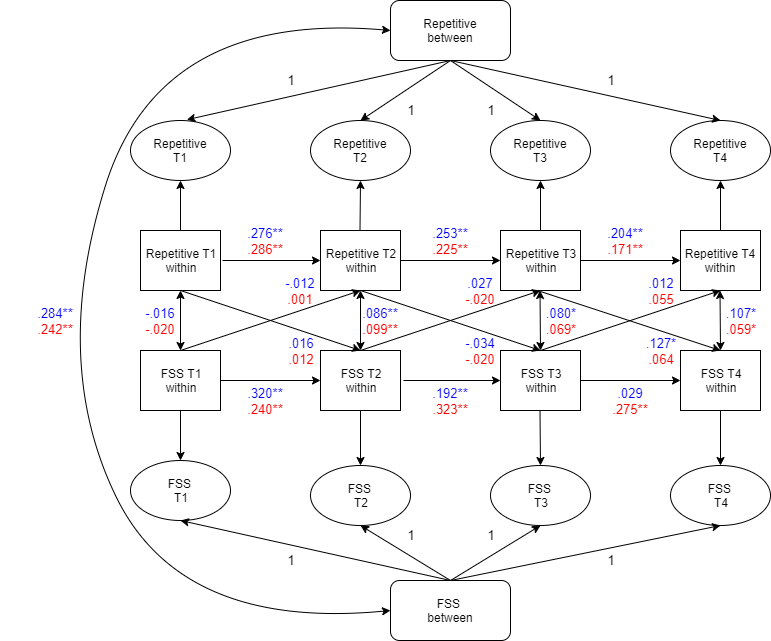


Note. * = significant at p < 0.05; ** = significant p < 0.01; coefficients in blue = male; coefficients in red = female

**Figure S3**. Summary of multi-group analyses in the optimal constrained RI-CLPM for self-regulatory behaviors and somatic symptoms.


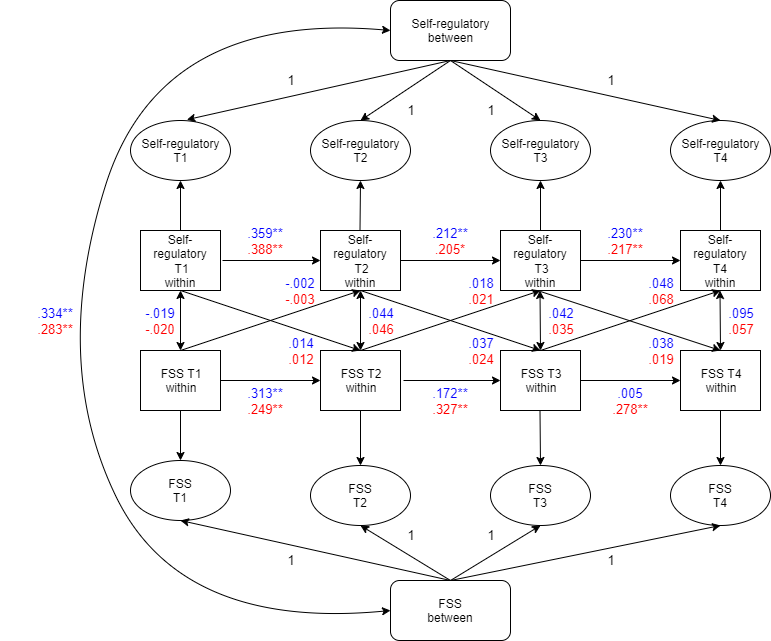


Note. * = significant at p < 0.05; ** = significant p < 0.01; coefficients in blue = male; coefficients in red = female

**Figure S4**. Summary of multi-group sensitivity analyses in the optimal constrained RI-CLPM in which all participants with chronic diseases were excluded.


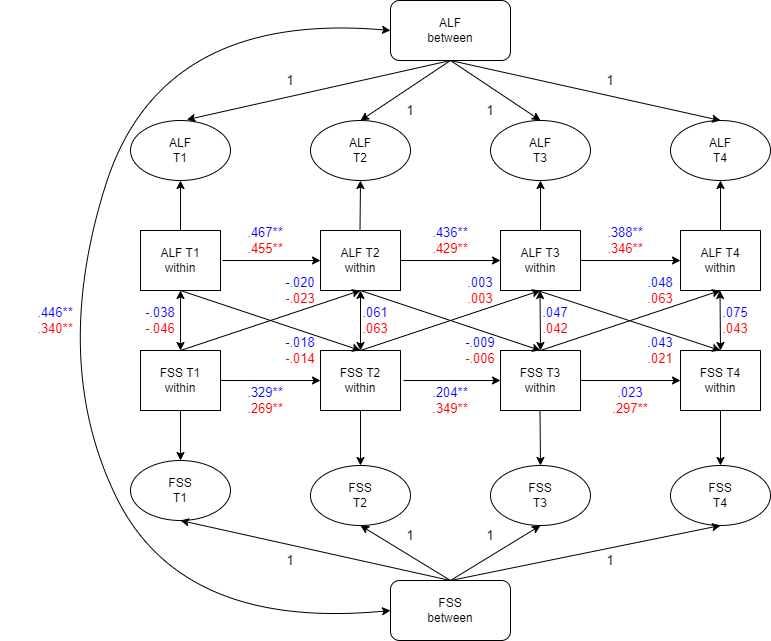


Note. * = significant at p < 0.05; ** = significant p < 0.01; coefficients in blue = male; coefficients in red = female; ALF = autistic-like features.

**Figure S5**. Summary of multi-group sensitivity analyses in the optimal constrained RI-CLPM in which cohort was added as covariate.


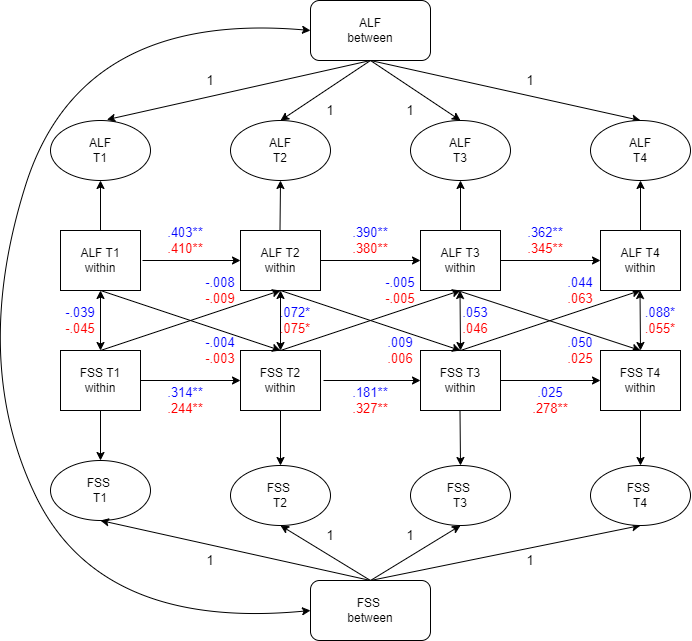


Note. * = significant at p < 0.05; ** = significant p < 0.01; coefficients in blue = male; coefficients in red = female; ALF = autistic-like features

**Figure S6**. Summary of multi-group sensitivity analyses in the optimal constrained RI-CLPM in a subsample of participants with a formal ASD diagnosis.

**
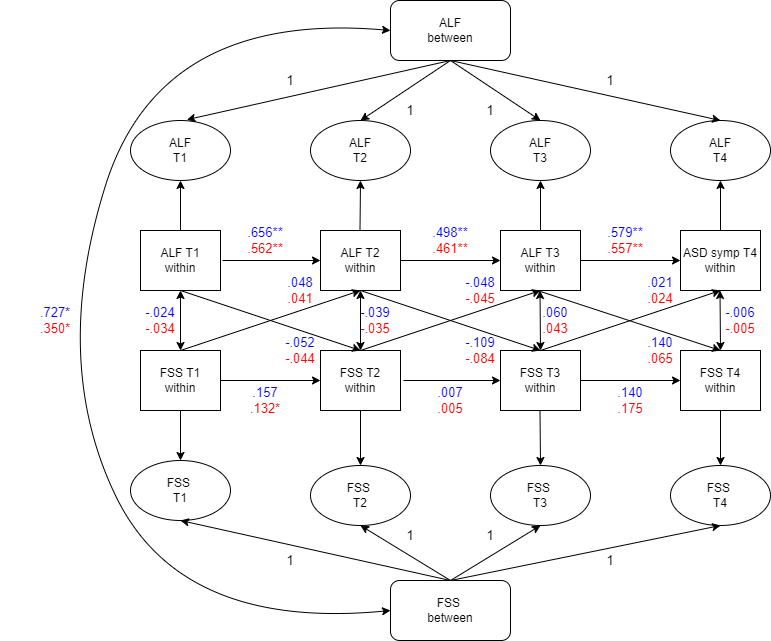
**

Note. * = significant at p < 0.05; ** = significant p < 0.01; coefficients in blue = male; coefficients in red = female; ALF = autistic-like features.

**Supplementary text 1**

**Main analysis**

At the within-person level, all autoregressive effects were significant, except for the path between FSS at T3 and FSS at T4 in males. These positive effects indicate that prior changes in autistic-like features predict changes in the same direction in autistic-like features at the subsequent wave, and likewise for FSS. Furthermore, we found small positive within-wave residual correlations at T2 (b = 0.003; after sex specific standardization: male: β = 0.069, *p* = 0.034; female: β = 0.073, *p* = 0.034) and at T4 (b = 0.003; after sex specific standardization: male: β = 0.096, *p* = 0.032; female: β = 0.058, *p* = 0.030) which demonstrate that, beyond the stable between-person correlation, within-person changes in autistic-like features were slightly positively related to within-person changes in FSS at the same wave.

**Supplementary text 2**

**Sensitivity analysis**

The within-wave residual correlations between autistic-like features and FSS at T2 and T4 now were non-significant (T2: b = 0.003; after sex specific standardization: male: β = 0.061, *p* = 0.071; female: β = 0.063, *p* = 0.071; T4: b = 0.002; after sex specific standardization: male: β = 0.075, *p* = 0.097; female: β = 0.043, *p* = 0.101). Besides that, parameter estimates were highly similar to those of the main analysis.

**Supplementary text 3**

**ASD subsample analysis**
By retrieving data from the PCRNN and psychiatric outpatient clinical healthcare files, we identified 290 participants with a clinical ASD diagnosis (244 of TRAILS-cc; 46 of TRAILS; 10.46% of the total sample). After deleting participants with missing data on autistic-like features and/or FSS on three or more waves, 264 participants were included in the subgroup analysis.

The within-wave residual correlations between autistic-like features and FSS at T2 and T4 now were non-significant (T2: b = -0.003; after sex specific standardization: male: β = -0.039, *p* = 0.679; female: β = -0.035, *p* = 0.675; T4: b = 0.000; after sex specific standardization: male: β = -0.006, *p* = 0.947; female: β = 0.005, *p* = 0.947). For males, none of the autoregressive effects of FSS were significant now (T1-T2: b = 0.156, β = 0.157, *p* = 0.059; T2-T3: b = 0.005, β = 0.007, *p* = 0.957; T3-T4: b = 0.143, β = 0.140, *p* = 0.239). For females, the autoregressive paths for FSS between T2 and T3, and between T3 and T4, now were non-significant (T2-T3: b = 0.005, β = 0.005, *p* = 0.957; T3-T4: b = 0.058, β = 0.175, *p* = 0.264). Besides that, parameter estimates were highly similar to those of the main analysis.
